# Metabolic Reprogramming in Amyotrophic Lateral Sclerosis

**DOI:** 10.1038/s41598-018-22318-5

**Published:** 2018-03-02

**Authors:** M. Szelechowski, N. Amoedo, E. Obre, C. Léger, L. Allard, M. Bonneu, S. Claverol, D. Lacombe, S. Oliet, S. Chevallier, G. Le Masson, R. Rossignol

**Affiliations:** 1 INSERM U1215, Neurocentre Magendie, 33077 Bordeaux, cedex France; 2 Bordeaux University, 33000 Bordeaux, France; 3 INSERM U1211, MRGM, 33000 Bordeaux, France; 4 CELLOMET, Center of Functional Genomics (CGFB), 146 Rue Léo Saignat, 33000 Bordeaux, France; 5 Center of Functional Genomics (CGFB), Proteomic Facility, Bordeaux University, 33000 Bordeaux, France

## Abstract

Mitochondrial dysfunction in the spinal cord is a hallmark of amyotrophic lateral sclerosis (ALS), but the neurometabolic alterations during early stages of the disease remain unknown. Here, we investigated the bioenergetic and proteomic changes in ALS mouse motor neurons and patients’ skin fibroblasts. We first observed that SODG93A mice presymptomatic motor neurons display alterations in the coupling efficiency of oxidative phosphorylation, along with fragmentation of the mitochondrial network. The proteome of presymptomatic ALS mice motor neurons also revealed a peculiar metabolic signature with upregulation of most energy-transducing enzymes, including the fatty acid oxidation (FAO) and the ketogenic components HADHA and ACAT2, respectively. Accordingly, FAO inhibition altered cell viability specifically in ALS mice motor neurons, while uncoupling protein 2 (UCP2) inhibition recovered cellular ATP levels and mitochondrial network morphology. These findings suggest a novel hypothesis of ALS bioenergetics linking FAO and UCP2. Lastly, we provide a unique set of data comparing the molecular alterations found in human ALS patients’ skin fibroblasts and SODG93A mouse motor neurons, revealing conserved changes in protein translation, folding and assembly, tRNA aminoacylation and cell adhesion processes.

## Introduction

Amyotrophic lateral sclerosis (ALS) is a rapidly progressive neurological disease characterized by the gradual degeneration and death of motor neurons, followed by global muscle wasting. Brain and systemic hypermetabolism have been observed in ALS patients, suggesting that energy-wasting mechanisms contribute to either ALS pathogenesis or adaptation to the disease. Numerous studies have investigated oxidative phosphorylation (OXPHOS) in different ALS models and revealed a global inhibition of the mitochondrial respiratory chain^1–5^. However, this does not explain energy wasting that rather suggests a defective coupling between mitochondrial respiration and ADP phosphorylation^6,7^ and/or a stimulation of the central metabolism and energy-transducing pathways. Uncoupling protein 2 (UCP2) was previously found to participate in mitochondrial reprogramming in ALS^3^. However, further bioenergetic and biochemical analyses showed that UCP2 does not uncouple OXPHOS as expected^8–14^ but rather accommodates a metabolic shift from glucose to fatty acid oxidation (FAO)^15^ and induce ketone body formation by exporting C4 metabolites as oxaloacetate out of mitochondria^16^. In line with this view, a metabolic shift toward FAO and ketogenesis was observed in the skeletal muscle of ALS patients^17^ and a genetic mouse model^4^, and UCP2 expression itself was shown to depend upon the activation of fatty acid metabolism^18^. Still, the bioenergetic and proteomic profiles of ALS presymptomatic motor neurons remain unknown, and the relative expression of glycolytic, OXPHOS and FAO enzymes has been underinvestigated. In conditions of hypermetabolism as it was extensively described in ALS, the mitochondrial respiratory chain require the sustained fueling of energy substrates to maintain a minimal electric membrane potential (Δψ) to avoid both a REDOX crisis and activation of apoptosis^19^. Given its higher yield in reductive equivalents compared with glucose oxidation, FAO could fulfill this requirement^15^. Moreover, in SOD1-related ALS cases or models, misfolded mutant SOD1 protein binds to and inhibit VDAC conductance at the mitochondrial outer membrane, thereby inducing bioenergetic blockade^2^. As fatty acids can diffuse through the mitochondrial outer membrane, FAO could by-pass this bioenergetic blockade. On the other hand, a long-term and chronic higher reliance on FAO could be toxic since FAO generates ketone bodies and ketoacidosis was shown to alter neurons viability in other metabolic pathologies such as in maple syrup urine disease and 2-methyl-3-hydroxybutyryl-CoA dehydrogenase deficiency.

Therefore, more detailed knowledge of the bioenergetics and proteome remodeling of presymptomatic motor neurons is required to derive adapted nutritional and pharmacological therapeutic strategies in ALS. Several lines of evidence indicate that motor neurons require OXPHOS^20–22^ for survival, and pro-OXPHOS protective metabolic approaches have been considered in clinical nutritional studies^23^. Recent studies have also revealed that during neuronal differentiation, the activation of OXPHOS is a critical checkpoint, and alterations in metabolic reprogramming impact neuronal survival^24–26^. However, the design of therapeutic approaches adapted to ALS neurometabolic specificities is hampered by our limited knowledge of motor neuron bioenergetics and metabolic reprogramming in ALS pathology. For instance, a systemic ketogenic diet could be deleterious if ALS motor neurons are already challenged, at the cellular level, by ketoacidosis secondary to adaptive FAO. While metabolic remodeling has been extensively described in cancer, diabetes, mitochondrial diseases and autoimmune disorders (for review see^27^), little information is available about this topic in neurodegenerative diseases such as ALS^28^. In the present study, we analyzed the bioenergetic alterations and the molecular changes in ALS mice motor neurons and patients’ skin fibroblasts. Our results unraveled the specificities of metabolic remodeling in ALS mouse motor neurons and patients’ skin fibroblasts.

## Results

### Bioenergetic alterations in presymptomatic SODG93A (ALS) mouse motor neurons

**Figure 1:**
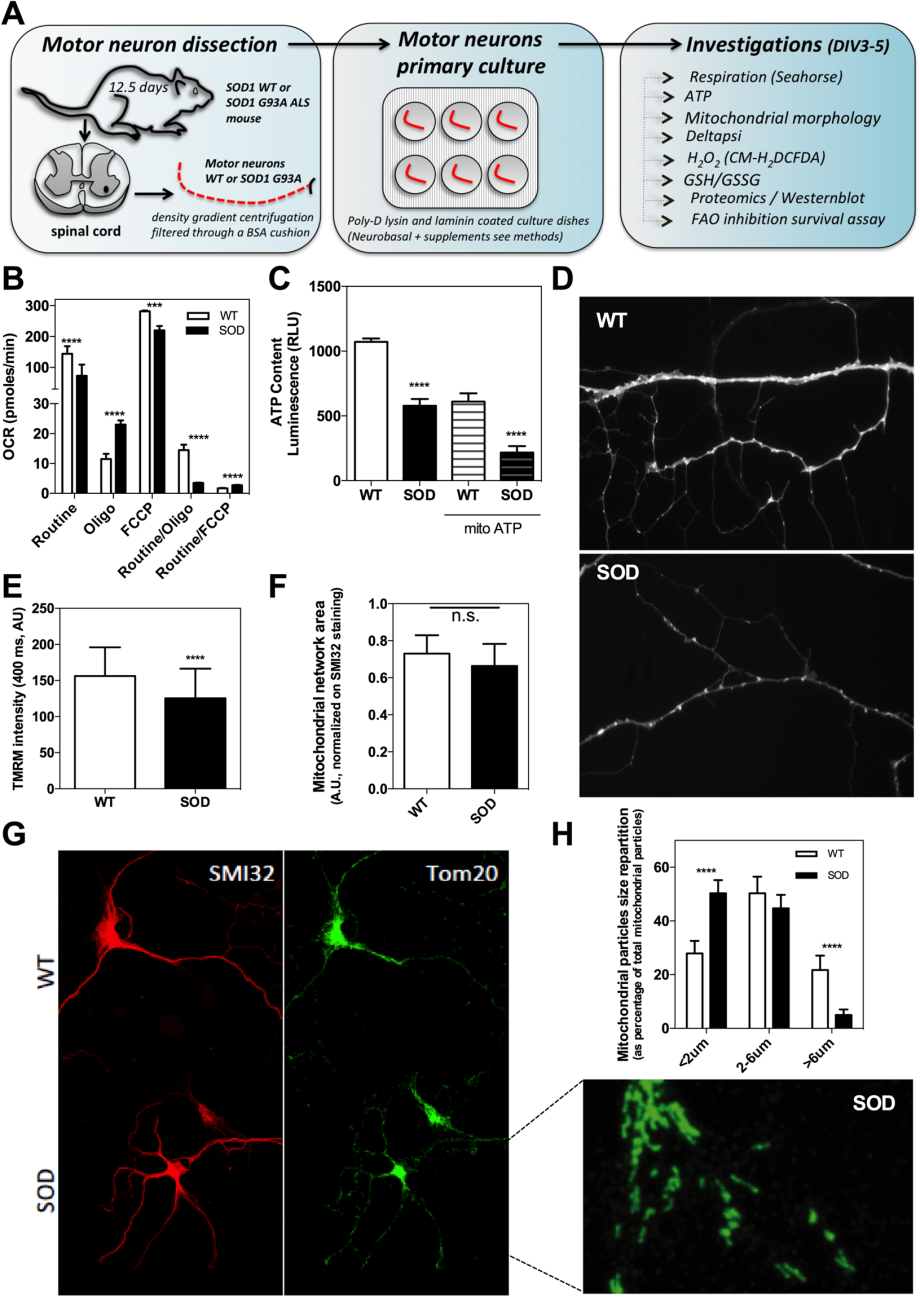
Bioenergetics of SOD1G93A ALS motor neurons. (**A**) Schematic workflow of the biochemical and cell biology investigations on presymptomatic mouse motor neurons at DIV3-5. (**B**) Oxygen consumption rate (OCR determined on the Seahorse XF96 extracellular flux analyzer) on SOD1G93A ALS mouse motor neurons (SOD) or wild-type mouse motor neurons (WT). Mitochondrial respiration was measured in routine, oligo or FCCP conditions as explained in the methods. **(C**) Total and mitochondrial steady-state ATP content in SOD or WT motor neurons. (**D**) Representative image of the TMRM signal for the measurement of mitochondrial transmembrane electric potential (Δψ) in WT or SOD mice motor neurons and (**E**) related quantification. (**F**) Mitochondrial network total area (TOM20 labelling) in mouse motor neurons normalized to the cellular area (SMI32 labeling) and corresponding images (**G**). (**H**) Mitochondrial network morphometric analysis in mouse motor neurons.

To gather a better understanding of the metabolic changes in presymptomatic ALS mice motor neurons, we performed a detailed bioenergetic and metabolic pathway proteome reprogramming analysis as summarized in Fig. 1A. First, respiration showed lower values in SODG93A mutant mice motor neurons than in wild-type ones under routine and FCCP (maximal respiration) conditions (Fig. 1B), as defined in the methods. A lower coupling degree of OXPHOS was also measured in ALS motor neurons, as determined by the reduced Routine/Oligo ratio (see methods). Accordingly, both the total cellular and the mitochondrial steady-state ATP levels were reduced in ALS motor neurons (Fig. 1C). The observed reduction in routine respiration was in agreement with the observed decrease in mitochondrial transmembrane electric potential (Δψ) measured in these conditions (Fig. 1D,E). The altered OXPHOS coupling in SODG93A mice motor neurons was not explained by usual uncoupling since routine respiration was not higher than in the wild-type mice motor neurons, and since respiration could be chemically uncoupled with Carbonyl cyanide-4-(trifluoromethoxy)phenylhydrazone (FCCP) **(**Fig. 1B). The FCCP/Routine ratio was even higher in SODG93A (ALS) motor neurons than in wild-type cells, indicating a large excess capacity of oxidative phosphorylation (Fig. 1B).

Therefore, our findings on ALS presymptomatic mouse motor neurons revealed *i)* an inhibition of mitochondrial respiration, *ii)* a reduction of OXPHOS coupling, *iii)* the non-occurrence of a pre-existing disease-related uncoupling, and *iv)* a lower steady-state mitochondrial electric transmembrane potential and cellular ATP level. Mitochondrial Δψ participates in the maintenance of organelle morphogenesis^29^, and significant alteration of the mitochondrial network was observed in motor neurons from ALS mice (Fig. 1F–H). In particular, a reduction in mitochondrial tubule size was observed, in agreement with the dependency of mitochondrial fusion on Δψ^30^. The mitochondrial network total area per motor neuron (mitochondrial density) was obtained as the ratio of the area of the TOM20 labeling to that of the SMI32 labeling. The data (Fig. 1H) indicated no significant difference in this parameter between WT and the ALS presymptomatic mouse motor neurons. Altogether, the investigation of mitochondrial physiology in ALS mice motor neurons revealed the occurrence of mitochondrial bioenergetic deficiencies and alterations in mitochondrial network architecture.

### UCP2 inhibition in ALS motor neurons alters bioenergetics but does not impact the redox status

**Figure 2:**
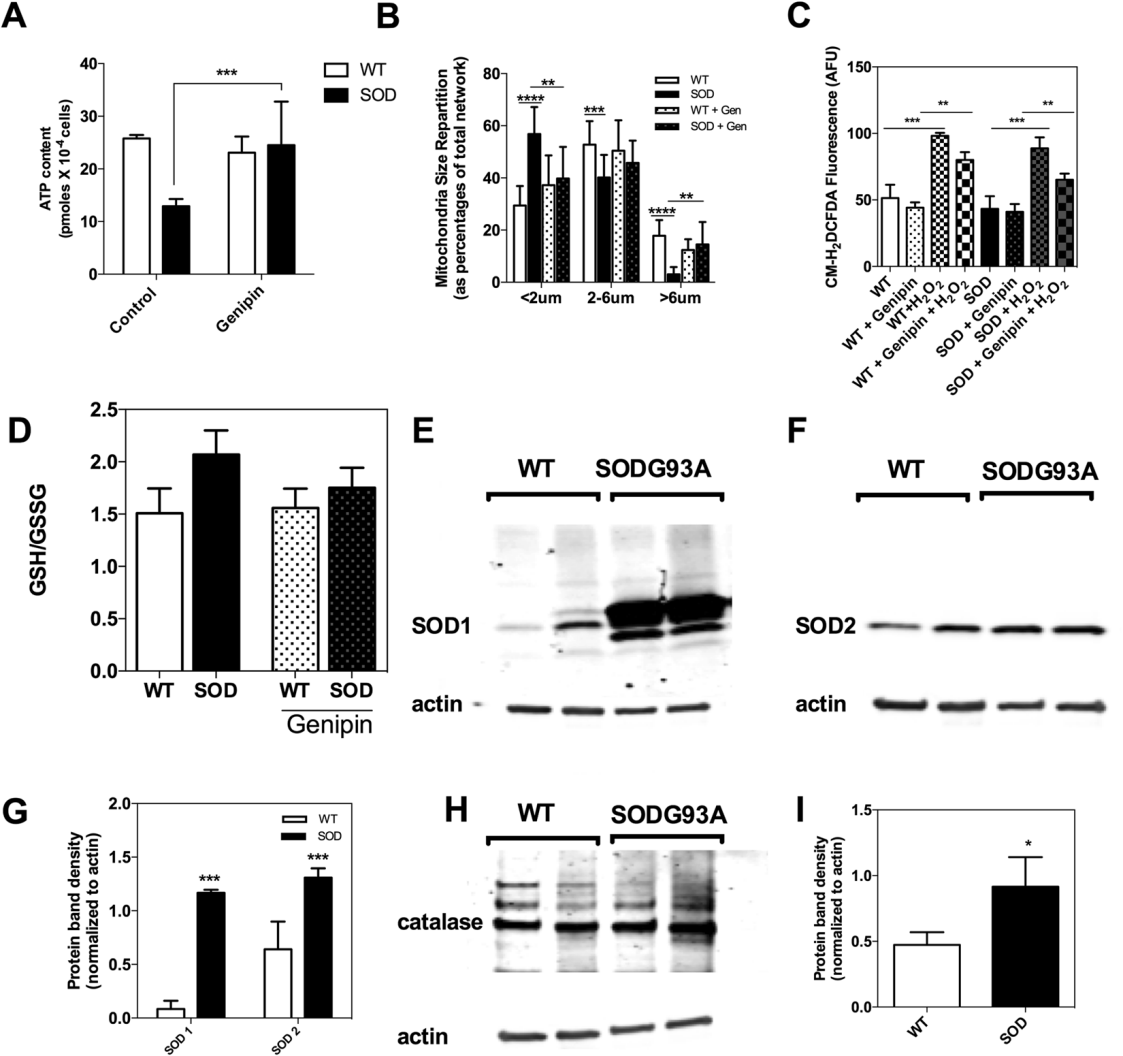
Role of UCP2 in ALS motor neurons bioenergetics and H_2_O_2_-scavenging. (**A**) Effect of UCP2 inhibition with genipin on motor neurons total ATP level, (**B**) mitochondrial network morphology, (**C**) H_2_O_2_ steady-state level (as measured by CM-H_2_DCFDA fluorescence) and (**D**) gluthathione redox state. (**E**) Westernblot analysis of the expression level of SOD1, (**F**) SOD2 and (**G**) relative quantification of SOD1 and SOD2 protein content (normalized to actin). (**H**) Westernblot analysis of the expression level of catalase and (**I**) relative quantification of catalase protein content (normalized to actin).

Previous investigations linked the uncoupling protein 2 (UCP2) to mitochondrial dysfunction in ALS^3,31,32^, so we tested the role of this protein in the bioenergetic alterations of ALS mice motor neurons observed in our study. Pharmacologic UCP2 inhibition with genipin induced a significant increase in the cellular ATP level (Fig. 2A) in ALS mice motor neurons and recovered the normal morphology of the mitochondrial network (Fig. 2B). However, no change in H_2_O_2_ level or ROS-scavenging capacity was triggered by genipin in both ALS and wild-type mice motor neurons in our experimental conditions (Fig. 2C). Likewise, the redox state of glutathione was not altered by UCP2 inhibition (Fig. 2D). Therefore, no direct sign of oxidative stress was found in presymptomatic ALS motor neurons, as neither the steady state level of H_2_O_2_ nor the GSH redox state showed differences between SODG93A and WT cells (Fig. 2C and D). However, a significant increase in antioxidant defense systems (SOD2 and catalase) was measured in the SODG93 A motor neurons by western blot (Fig. 2E–I), suggesting an indirect sign of oxidative stress. These findings indicate that in ALS motor neurons, UCP2 participates in ATP synthesis as recently proposed^16^ and not act as an uncoupling protein.

### Proteome remodeling in presymptomatic motor neurons from ALS mice

**Figure 3:**
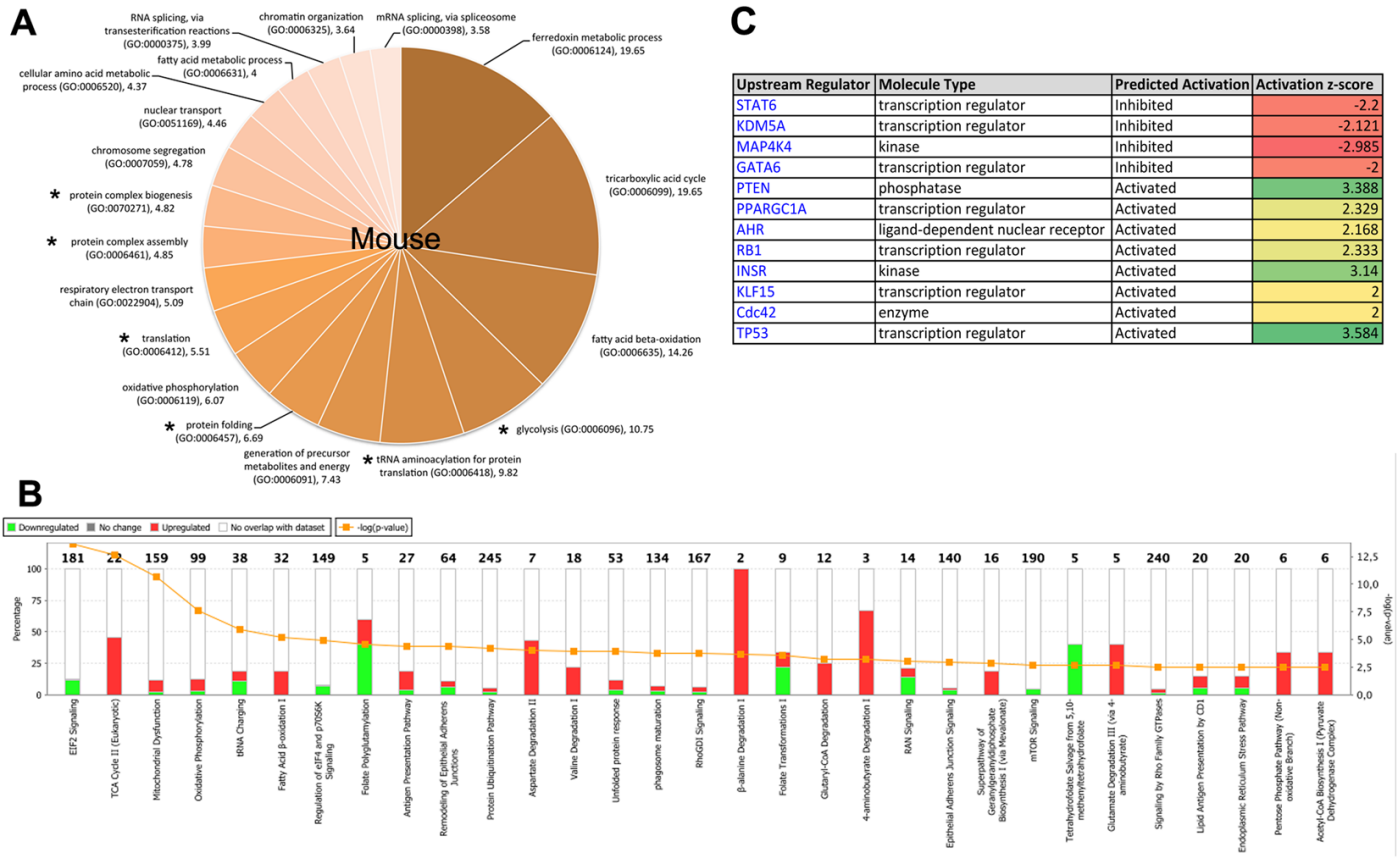
SODG93A ALS mouse motor neurons proteome. **(A)** Over-represented GO terms in the proteome of ALS mouse motor neurons as compared to the mouse reference transcriptome. (**B**) Comparison of the proteome of SODG93A ALS mice motor neurons and that of wild-type mice motor neurons. The Ingenuity Pathways Analysis was performed to identify the pathway significantly different (p < 0.05) between the two proteomes. The −log p value is shown in the orange line and in the bottom abscissa. The percentage of proteins from the pathway found in the differential proteome is given in the top abscissa. (**C**) Table of the predicted main regulators of the ALS mouse motor neurons proteome remodeling.

To investigate the broad impact of the SODG93A mutation in mice motor neurons, we performed a large-scale molecular study using label-free proteomics. Firstly, we evaluated the cellular functions (Gene Ontology term (GO terms)) over-represented in the differential proteomic dataset. The results indicated that energy-transducing enzymes belonging to several pathways such as FAO, the TCA cycle, glycolysis and OXPHOS were increased (Fig. 3A**)**. In addition, different functions linked with protein translation, folding or complex assembly were also over-represented. Likewise, over-representation of proteins implicated in mRNA splicing, chromosome segregation and chromatin organization was observed in presymptomatic ALS mouse motor neurons.

Secondly, we compared the proteome of SODG93A and WT mouse motor neurons. This analysis identified 496 proteins that differed in content between the two groups of motor neurons (ALS vs WT, p < 0.05). A total of 290 proteins were over-represented in ALS (fold change ranging from 1.12 to >1000), and 206 were repressed. A global analysis of the cellular functions impacted by those changes, taking into account both the over- or under-expressed proteins with a cut-off of –Log(*p* value) >2.5, was performed using Ingenuity Pathway Analysis **(**IPA, Fig. 3B**)**. The top five IPA pathways that differ between ALS and WT motor neurons were ‘EIF2 signaling (p = 2.61 E-14)’, ‘TCA cycle (p = 2.14 E-13)’, ‘mitochondrial (dys)function (p = 2.11 E-11)’, ‘oxidative phosphorylation (p = 2.29 E-8)’ and ‘tRNA charging (p = 1.15 E-6)’. Of note, the ‘superpathway of cholesterol biosynthesis (via mevalonate)’ was also upregulated in SOD motor neurons with −log p = 2.82.

**Figure 4:**
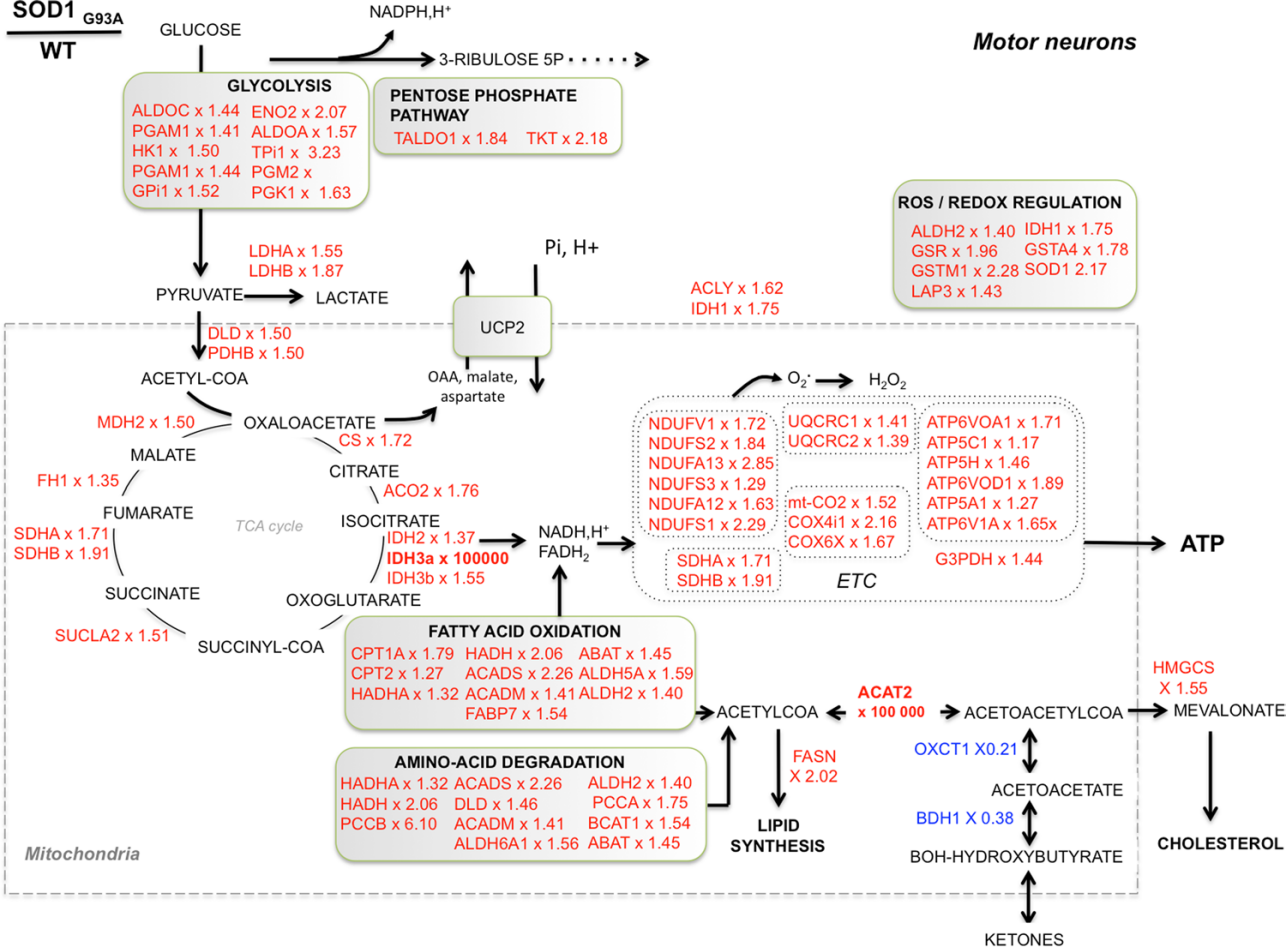
Proteome remodeling in SODG93A ALS motor neurons. Chart of the metabolic proteome remodeling in SODG93A ALS motor neurons. Over represented proteins appear in red and the corresponding fold change (SOD/WT) is given after the name of each protein.

The top two protein networks significantly different between ALS and WT mouse motor neurons (Supplemental Figure S1A and B, respectively) were ‘protein synthesis, protein degradation cellular assembly and organization’ (score 38)’ and ‘lipid metabolism, small molecule biochemistry, energy production’ (score 28). The search for predicted regulators of the observed proteomic differences in ALS *versus* WT motor neurons identified 4 inhibited factors (p < 0.05) and 8 activated ones (Fig. 3C). The proteome analysis also suggested that neuritogenesis and formation of cellular protrusions could be activated in ALS mice motor neurons based on the increased expression of specific proteins involved in theses functions (Supplemental Table S1). The predicted alteration (activation Z score > 2 or <−2) of other cellular functions as ‘cell death’, ‘organization of the cytoskeleton’ ‘microtubule dynamics’, ‘fragmentation of DNA’ and ‘concentration of fatty acids’ was also detected in this analysis (Supplemental Table S1). Altogether, the proteomic survey indicated an important modification of the metabolic machinery in motor neurons from ALS mice, with a particular enhancement of the molecular systems involved in oxidative energy metabolism. The Kyoto Encyclopedia of Genes and Genomes (KEGG) pathway analysis of the proteins over-represented in the ALS motor neuron proteome provided a more detailed description of the changes in the canonical metabolic pathways (Supplemental Table S2**)**. It showed a large number of differences (FDR < 0.05) in energy-transducing pathways such as OXPHOS, TCA cycle, branched-chain amino-acids degradation, propanoate metabolism, glycolysis and fatty acid oxidation. A metabolic chart of those changes is shown in Fig. 4, demonstrating the major differences in the mitochondria, with a dramatic increase in ACAT2 (ketone metabolism). In addition to energy metabolism, lysosome, phagosome and protein processing in the ER were also observed among the over-represented KEGG pathways in ALS mice motor neurons (Supplemental Table S2). Lastly, the KEGG analysis of the proteins under-represented in ALS mice motor neurons (Supplemental Table S3) showed a significant (FDR < 0.05) alteration of the ‘ribosomes’, ‘spliceosome’ and components belonging to the ‘protein processing in endoplasmic reticulum’ category. A detailed analysis of the changes in ribosomal proteins is shown in Table 1. Two proteins involved in the synthesis and degradation of ketone bodies were also identified in the under-represented KEGG pathways, namely 3-hydroxybutyrate dehydrogenase 1 (BDH1) and 3-oxoacid-CoA transferase 1 (OXCT1). Lastly, the KEGG analysis of the under-represented pathways in ALS mice motor neurons (Supplemental Table S3) identified gap junction, focal adhesion and tight junction, suggesting a possible alteration of the cell adhesion processes of motor neurons.

**Table 1:** Detail of the proteomic changes in ribosomal subunits and in components of the protein processing in the ER system in ALS mice motor neurons and ALS patients skin fibroblasts.

Decreased proteostasis components in ALS
RS2_HUMAN, **RS3_HUMAN**, RS3A_HUMAN, RS4X_HUMAN, RS4Y1_HUMAN, RS4Y2_HUMAN, *RS5_HUMAN*, *RS6_HUMAN*, RS7_HUMAN, RS8_HUMAN, RS9_HUMAN, RS10_HUMAN, *RS11_HUMAN*, RS12_HUMAN, RS13_HUMAN, **RS14_HUMAN**, *RS15_HUMAN*, RS15A_HUMAN, *RS16_HUMAN*, RS17_HUMAN, RS18_HUMAN, RS19_HUMAN, RS20_HUMAN, RS21_HUMAN, **RS23_HUMAN**, RS24_HUMAN, RS25_HUMAN, RS26_HUMAN, *RS27_HUMAN*, RS28_HUMAN, RS29_HUMAN, RS30_HUMAN, RSSA_HUMAN, Gm6336, Gm2000, Gm6139, SBDS, RAN, Gtpbp4, RL3_HUMAN, RL4_HUMAN, *RL5_HUMAN*, RL6_HUMAN, *RL7A_HUMAN*, RL7_HUMAN, **RL8_HUMAN**, RL9_HUMAN, RL10A_HUMAN, **RL11_HUMAN**, RL12_HUMAN, RL13A_HUMAN, RL14_HUMAN, RL15_HUMAN, RL17_HUMAN, RL18A_HUMAN, RL19_HUMAN, RL21_HUMAN, RL22_HUMAN, RL23A_HUMAN, *RL23_HUMAN*, RL24_HUMAN, RL26_HUMAN, RL27A_HUMAN, RL27_HUMAN, RL28_HUMAN, RL29_HUMAN, RL30_HUMAN, RL31_HUMAN, RL32_HUMAN, RL34_HUMAN, RL35A_HUMAN, RL35_HUMAN, RL36A_HUMAN, RL36_HUMAN, RL37A_HUMAN, RL37_HUMAN, RL38_HUMAN, RL39_HUMAN, RL40_HUMAN, RL41_HUMAN, RLA0_HUMAN, RLA1_HUMAN, RLA2_HUMAN.
**Increased proteostasis components in ALS**
CANX, **HSPA5**, MOGS, HYOU1, PRKCSH, CKAP4, **DDOST**, UGGT1, **PDIA3, CALR**, RPLP1, RPL24, RPS8, Capn2, Vcp, **Hspa5**, Lman2, **Ddost, Pdia3**, Ganab, Pdia4, **Calr**, Rpl19.

This table was obtained from the Kyoto Encyclopedia of Genes and Genomes (KEGG) analysis of the proteomic datasets obtained on ALS mouse motor neurons and in ALS patient’s skin fibroblasts. The subunits altered in skin only are written in italic, the subunit altered in motor neurons only are in normal case and all the ones altered in both skin and motor neurons are in bold.

### OXPHOS decoupling and proteome remodeling in skin fibroblasts from ALS patients

**Figure 5:**
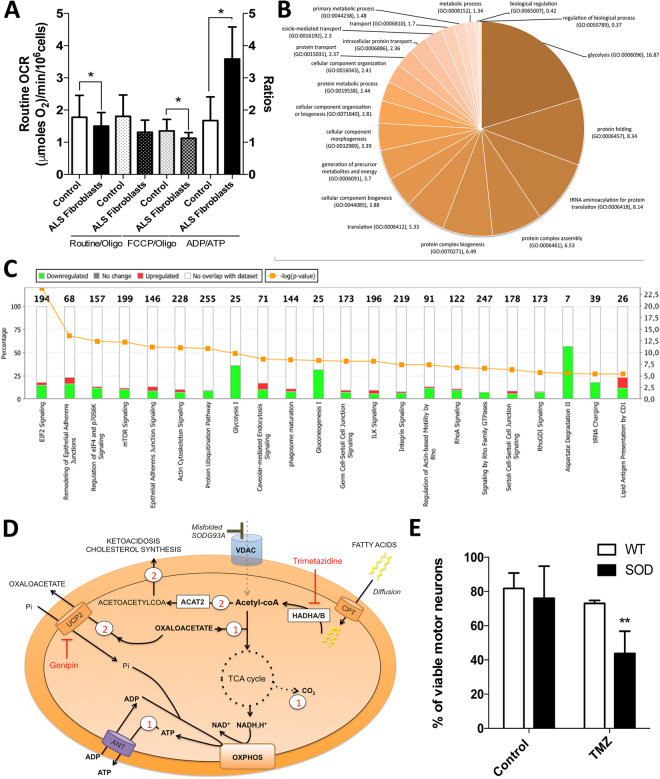
Bioenergetic alterations and proteome remodeling in ALS patient skin fibroblasts. **(A)** Respiration of skin fibroblasts in routine conditions and calculation of the respiratory control ratio (routine/oligo respiration) and of the uncoupling ration (FCCP/oligomycin respiration). (**B**) ADP/ATP ratio determined in skin fibroblasts. (**C**) Proteome analysis (over-representation test) of ALS skin patients fibroblasts. (**D**) Comparison of the proteome between ALS skin patients fibroblasts and fibroblasts from healthy individuals. Ingenuity Pathway Analysis (IPA) was performed and the pathway with a difference of −log *p*value > 5 are shown. (**E**) Bioenergetic model of ALS adaptive metabolism showing the role of HADHA, ACAT2, UCP2 and HMGCS1. In this hypothetical model, ALS motor neurons shift to FAO and amino-acids degradation to transduce energy but in turn generate acetyl-CoA which can then be used for fatty-acid synthesis, ketogenesis and cholesterol synthesis. Therefore, a fine tuning between the use of FAO and the elimination of acetyl-CoA must be maintained to promote cell survival, and UCP2 could play a central role in this balance. In our model, the use of acetyl-coA produced by FAO for either oxidative phosphorylation (route 1) or ketogenesis and cholesterol synthesis (route 2) depends on the export of oxaloacetate by UCP2 (route 2), as proposed by Vozza *et al*.^16^. In line with this hypothesis, (i) the inhibition of UCP2 with genipin leads to an increase in cellular ATP levels (blockade of route 1; Fig. 2A), while ii) the blockade of FAO using trimetazidine (HADHB inhibitor) reduces cell viability in SOD motor neurons (blockade of route 1), with minor effects on wild-type cells (Fig. 5E). This model considers previous findings indicating that VDAC is blocked by mutant-SOD in ALS^2^, forcing cells to depend on outer membrane-diffusive energy sources such as fatty acids. It also considers recent findings showing the accumulation of cholesterol in the cerebrospinal fluid of ALS patients^33^.

Similar to mice motor neurons, we compared mitochondrial bioenergetics and proteome composition in human skin fibroblasts taken from ALS patients (Supplemental Table S4**)** with controls (male 58 years old and female 46 years old). Respiration analysis first showed a reduced OXPHOS coupling, as assessed by a reduced effect of oligomycin, without changes in routine respiration (Fig. 5A). This reduction in OXPHOS coupling was associated with a corresponding decrease in the steady-state ATP level and an increase in ADP content in ALS patients’ skin fibroblasts (Fig. 5A). The uncoupling ratio (FCCP-stimulated respiration/oligomycin-treated respiration) was also reduced in the patient’s fibroblasts as compared to the controls (Fig. 5A).

The over-representation test performed on the proteome of ALS skin fibroblasts (Fig. 5B) showed enrichment in the altered expression of proteins of glycolysis, translation, protein folding, protein complex biogenesis, protein complex assembly, and tRNA aminoacylation. To better analyze the proteome specificities of ALS human skin fibroblasts, we performed a comparison with skin fibroblasts obtained from age-matched healthy individuals. This comparison identified 115 over-represented and 262 under-represented proteins in ALS skin fibroblasts. The Ingenuity Pathway Analysis (Fig. 5C) indicated major changes in ‘EIF2 signaling’, ‘TCA cycle’, ‘oxidative phosphorylation’, ‘fatty-acid oxidation’, ‘remodeling of epithelial adherens junctions’, and ‘glycolysis’ (see complete list in Table S5). A more detailed search for the alterations in cellular metabolic pathways (using the ‘toxicology’ module of IPA) showed modification of ‘fatty acid metabolism’, ‘NRF2-mediated oxidative stress response’ and ‘PPARα/RXRα pathway activation (p < 0.05)’. The main predicted regulators of the observed proteomic changes are listed in Supplemental Table S6. In particular, IPA predicted the activation of three transcription factors (TFAP2A, JUNB, and PDX1) and the inhibition of five others (HSF1 and HSF2 involved in the proteotoxic stress response, NFE2L2 involved in the antioxidant response and, MYCN and MYC involved in cellular reprogramming). Regarding MYC, 85 targets of this transcription factor were found in the difference proteome (Supplemental Figure S2). Activation of the mTORC2 central component RICTOR was also predicted by IPA (32 target genes; Supplemental Figure S2).

The KEGG analysis of the proteins over-represented (FDR < 0.05) in ALS patients’ skin fibroblasts revealed enrichment in ‘protein processing in endoplasmic reticulum’, ‘focal adhesion’ and ‘tight junctions’ (Table 2). Concerning the changes in metabolic pathways, the two subunits of the mitochondrial trifunctional protein complex (HADHA and HADHB) involved in fatty acid oxidation were overexpressed. Another related major change in ALS mice motor neurons was the expression of ACAT2, a protein involved in the reversible production of ketones from the main FAO final product: acetyl-coA. Lastly, the analysis of the proteins under-represented in the ALS patients’ skin fibroblasts revealed a significant (FDR < 0.05) reduction in components of glycolysis, ribosome, focal adhesion, proteasome, RNA transport, fatty-acids degradation and pentose phosphate pathway (Table S7).

**Table 2:** Kyoto Encyclopedia of Genes and Genomes (KEGG) pathway analysis of the over- and under-expressed components in ALS (P < 0.05; at least 2 proteins per pathway). FDR (False discovery Rate).

Pathway description	FDR	Overexpressed proteins in ALS patient’s fibroblasts
Protein processing in endoplasmic reticulum	6.89E-08	CALR,CANX,CKAP4,DDOST,HSPA5,HYOU1,MOGS,PDIA3,PDIA4,PRKCSH,UGGT1
Amoebiasis	7.86E-05	ACTN1,ACTN4,COL1A2,COL5A1,FN1,GNAQ,RAB7A
Focal adhesion	0.000439	ACTN1,ACTN4,COL1A2,COL5A1,FLNA,FLNB,FN1,ITGAV
Thyroid hormone synthesis	0.00133	CANX,GNAQ,GPX8,HSPA5,PDIA4
Salmonella infection	0.00216	FLNA,FLNB,MYH10,MYH9,RAB7A
Regulation of actin cytoskeleton	0.00252	ACTN1,ACTN4,FN1,GSN,ITGAV,MYH10,MYH9
Antigen processing and presentation	0.0103	CALR,CANX,HSPA5,PDIA3
Tight junction	0.0113	ACTN1,ACTN4,CTNNA1,MYH10,MYH9
Arrhythmogenic right ventricular cardiomyopathy	0.0119	ACTN1,ACTN4,CTNNA1,ITGAV
ECM-receptor interaction	0.0173	COL1A2,COL5A1,FN1,ITGAV
Protein digestion and absorption	0.0173	COL12A1,COL1A2,COL5A1,SLC3A2
Lysine degradation	0.0388	GLT25D1,HADHA,PLOD3

### ALS motor neuron survival relies on FAO

The proteomic analyses described above indicated that several components of the fatty-acids oxidation system were upregulated both in ALS mouse motor neurons and ALS patients’ skin fibroblasts. Conversely, the ketone bodies metabolizing enzymes BDH1 and OXCT1 were repressed while ACAT2 was strongly upregulated as well as the cytoplasmic hydroxymethylglutaryl-CoA synthase involved in cholesterol synthesis 1 (see Fig. 4). Moreover, we found that UCP2 activity was linked with energy homeostasis in ALS motor neurons (Fig. 2A), which is consistent with UCP2 role in exporting oxaloacetate from mitochondria. Lastly, the biochemical survey of lipid metabolism performed in ALS patients indicated ketonuria in three out of the five patients investigated (Supplemental Table S4). Taken together, these proteomic and bioenergetic findings could suggest a working model of ALS motor neurons metabolic rewiring as illustrated in Fig. 5D. In this hypothetical model, ALS motor neurons shift to FAO and amino acids degradation to transduce energy but in turn generate acetyl-CoA, which can then be used for fatty-acid synthesis, ketogenesis and cholesterol synthesis. Therefore, a fine tuning between the use of FAO and the elimination of acetyl-CoA must be maintained to promote cell survival, and UCP2 could play a central role in this balance. In our model, the use of acetyl-coA produced by FAO for either oxidative phosphorylation (route 1) or ketogenesis and cholesterol synthesis (route 2) depends on the export of oxaloacetate by UCP2, as proposed by Vozza *et al*.^16^. In line with this hypothesis, (i) the inhibition of UCP2 with genipin leads to an increase in cellular ATP levels (blockade of route 1; Fig. 2A), while ii) the blockade of FAO using trimetazidine (HADHB inhibitor) reduces cell viability in SOD motor neurons (blockade of route 1), with minor effects on wild-type cells (Fig. 5E). This model considers previous findings indicating that VDAC is blocked by mutant-SOD in ALS^2^, forcing cells to depend on mitochondrial outer membrane-diffusive energy sources such as fatty acids. It is also consistent with recent observations of cholesterol accumulation in the cerebrospinal fluid of ALS patients^33^.

## Discussion

Alteration of energy metabolism is considered to be a hallmark of amyotrophic lateral sclerosis pathophysiology^34^ since multiple lines of evidence have shown inhibition of respiratory chain enzymes complex activity in patients’ muscle^35^ and spinal cord^36^ samples, as well as in tissues of ALS mice models^37,38^. Still, little is known about the bioenergetics and proteomic changes in ALS motor neurons during the early stages of the disease.

In the present study, we analyzed the bioenergetics and molecular reprogramming of embryonic presymptomatic motor neurons from SODG93A ALS mice at 2 to 5 DIV. We found a defective oxidative phosphorylation system, characterized by reduced respiration and lower coupling, which accentuates ATP depletion. The inhibition of the respiratory chain system was previously explained by the blockade of VDAC by the misfolded mutated form of SOD1^2^, and the altered OXPHOS coupling was thought to result from the perturbation of mitochondrial structure^39^. Accordingly, we observed an increased fragmentation of the mitochondrial network in ALS mice motor neurons, as well as a reduced mitochondrial transmembrane electric potential. This is totally consistent with the dependency of mitochondrial morphology and dynamics on mitochondrial bioenergetics^29^ and on transmembrane electric potential^30^. Our findings demonstrate that oxidative phosphorylation is not uncoupled in ALS but rather decoupled since the ADP-stimulated rate of respiration was reduced (lower effect of oligomycin) while the non-phosphorylating respiratory rate was not increased and was sensitive to the effect of uncouplers. The difference and theoretical background between uncoupling and decoupling was extensively discussed by Rottenberg H.^40^. Briefly, OXPHOS uncoupling is characterized by a reduced mitochondrial transmembrane electrochemical gradient of protons associated with an increased proton permeability and reduced respiratory rate, while decoupling is characterized by a reduction of the ADP-stimulated rate of respiration without increase in non-phosphorylating rate of respiration.

Our molecular investigations revealed that ALS mutant motor neurons, with defective bioenergetics, have developed adaptive strategies to survive. Indeed, using comprehensive label-free proteomics, we found that most pathways involved in glucose, fatty- acid, and amino acid catabolism are up-regulated in ALS mice motor neurons, while only amino-acid degradation pathways and a step of fatty-acid oxidation are enhanced in patients’ skin fibroblasts. In particular, our study revealed a potential biomarker that is increased in both ALS mice motor neurons and patients’ skin fibroblasts: HADHA, the trifunctional enzyme complex subunit alpha located in the mitochondria, also known as hydroxyacyl-CoA dehydrogenase/3-ketoacyl-CoA thiolase/enoyl-CoA hydratase. Validation of this potential biomarker will require investigation in a larger group of ALS patient samples as recently performed for the urinary extracellular domain of the neurotrophin receptor p75-ECD^41^.

While numerous studies have indicated that neuronal bioenergetics depends primarily on glucose catabolism *via* the neuron-glia metabolic symbiosis, an NMR study performed in adult rat brain showed that approximately 20% of the total energy was obtained from fatty acids^42^. Our findings demonstrated that ALS mice motor neurons rely directly on this pathway for survival. More generally, enhanced expression of β-oxidation enzymes features a bioenergetic adaptive process in situations of metabolic stress^43^, as it was already proposed in idiopathic Parkinson’s disease^44^. Still, FAO generates toxic end-products as ketone bodies, and a bioenergetic shift toward FAO could have implications for neuron viability if ketoacidosis occurred. Accordingly, another protein strongly over-represented in ALS mice motor neurons was acetyl-CoA acetyltransferase 2 (ACAT2), which catalyzes the reversible conversion of two units of acetyl-CoA to acetoacetyl CoA (ketogenesis) or the thiolytic cleavage of 3-ketoacyl-CoA into two-carbon chain-shortened acyl*-*CoA plus acetyl*-*CoA (ketosis). In the context of an increased expression of fatty-acid oxidation enzymes (notably the mitochondrial trifunctional protein), ACAT2 over-expression may facilitate the removal of the FAO end-product acetyl-coA, to produce acetoacetyl CoA. Given that ALS motor neurons display a strongly reduced expression of two enzymes involved in ketone bodies formation and utilization, namely 3-hydroxybutyrate dehydrogenase 1 (BDH1) and 3-oxoacid-CoA transferase 1 (OXCT1), one could hypothesize that the acetoacetyl-CoA could be used for cholesterol synthesis by the mevalonate pathway. This view is supported by the increased expression of the HMG-CoA-Synthase 1 that we observed in ALS mouse motor neurons (see Fig. 4) and the recent discovery of increased levels of cholesterol in the CSF of ALS patients^33^. However, we cannot exclude a role of ACAT2 in ketosis and OXPHOS fueling, and this point will require further metabolic investigations.

We did not find any evidence of downregulation of respiratory chain subunits in the proteome of ALS skin fibroblasts but we discovered a downregulation of glucose metabolism (glycolysis) and malate-aspartate shuttle components (GOT1, GOT2, MDH2) suggesting a reduced fueling of OXPHOS rather than a decreased biogenesis of the respiratory chain. Conversely, the proteome of ALS motor-neurons showed a strong increase in the expression of respiratory chain proteins indicative of a compensatory response to the initial OXPHOS defect secondary to SOD1 mutation, as proposed by Israelson, A *et al*.^2^.

Taken together, our bioenergetic and proteomic findings allow us to propose a working model of ALS motor neuron metabolic rewiring linking UCP2, HADHA, ACAT2 and HMGCS1 (see Fig. 5D). In this hypothesis, the export of oxaloacetate by UCP2^16^ lies at the crossroad between, on the one hand, fatty-acid-supported and TCA cycle-dependent ATP synthesis and, on the other hand, ketogenesis and cholesterol synthesis. The role of UCP2 expression in fatty acid oxidation, ketogenesis and cholesterol synthesis has been suggested in previous studies^8,45,46^, and our findings support the role of UCP2 in the regulation of cellular ATP levels rather than ROS scavenging. Moreover, HADHA, ACAT2, UCP2 and HMGCS1 are all positively regulated by PPARα, and a recent study in SODG93A ALS mice showed that PPARα transcriptional activity is increased in the spinal cord^47^. The fifth protein of interest in our study was isocitrate dehydrogenase isoform 3, which was strongly upregulated in ALS motor neurons (top protein with ACAT2). IDH3 differs from IDH1 and IDH2 in that it reduces NAD^+^, while IDH1 and IDH2 use NADP^+^ as a co-factor. Moreover, IDH1 and IDH2 are reversible and can participate in the reductive carboxylation process involved in *de novo* fatty acid biosynthesis, in contrast with IDH3. Therefore, the overexpression of IDH3 in presymptomatic ALS mouse motor neurons could further facilitate the metabolic shift toward FAO.

The proteomic study of ALS motor neurons revealed significant changes in protein synthesis and degradation, which is in agreement with previous reports showing that altered proteostasis in ALS represents one of the earliest pathological signature of the disease^48^. Proteostasis is a high energy-demanding process, and ER stress alone can activate the upregulation of energy transducing proteins via mTOR inhibition and AMP-activated protein kinase (AMPK) activation^49^. Furthermore, AMPK activation correlates with ALS progression in mutant SOD1 mice motor neurons^50,51^. Interestingly, reducing AMPK activity pharmacologically or genetically prevents mutant SOD1G93A-induced motor neuron death *in vitro*^52^, suggesting that although AMPK activation promotes energy homeostasis at early stages of neuron differentiation, it could also participate in the onset of progressive neurodegeneration. This view is in accordance with our hypothesis of ‘toxic-FAO’ in motor neurons, as AMPK stimulates FAO but also promotes ketone body formation and cholesterol synthesis^53^.

ALS is characterized by the accumulation of protein aggregates that affect the function of motor neurons, and changes in ribosome composition and function might be expected. We found a set of ribosomal proteins and some components of protein processing in the ER that similarly varied in ALS mouse motor neurons and in ALS patients’ skin fibroblasts: 40S ribosomal proteins S3, S14, and S23 and 60 S ribosomal proteins L8 and L11 were down-regulated, while 78 kDa glucose-regulated protein (HSP5A), dolichyl-diphosphooligosaccharide-protein glycosyltransferase 48 kDa subunit, protein disulfide-isomerase A3 and calreticulin were up-regulated in both models. Those proteins participate in the protein processing module in the ER, which includes components of the ER-associated degradation (ERAD) and components of the unfolded protein response (UPR). In addition to ribosomes and protein processing, we observed a down-regulation of proteins involved in cell adhesion and intercellular contacts in ALS mouse motor neurons. However, little is known about such changes in motor neurons in ALS patients. Recently, Krieger *et al*. questioned the relevance of molecules mediating cell adhesion in the pathogenesis of ALS, which might be important at the neuromuscular junction, since detachment of the synapses between the motor neuron and the skeletal muscle fibers has been observed^54^. Altogether, our findings on the ALS mice motor neuron proteome could designate protein candidates of interest for future biochemical and preclinical studies in the area of energy metabolism, protein translation and cell adhesion. Furthermore, the large-scale proteome remodeling observed in ALS motor neurons could be linked to the bioenergetic alterations described in our study, since metabolic checkpoints were identified as crucial steps during neuronal differentiation^24–26^.

**Figure 6:**
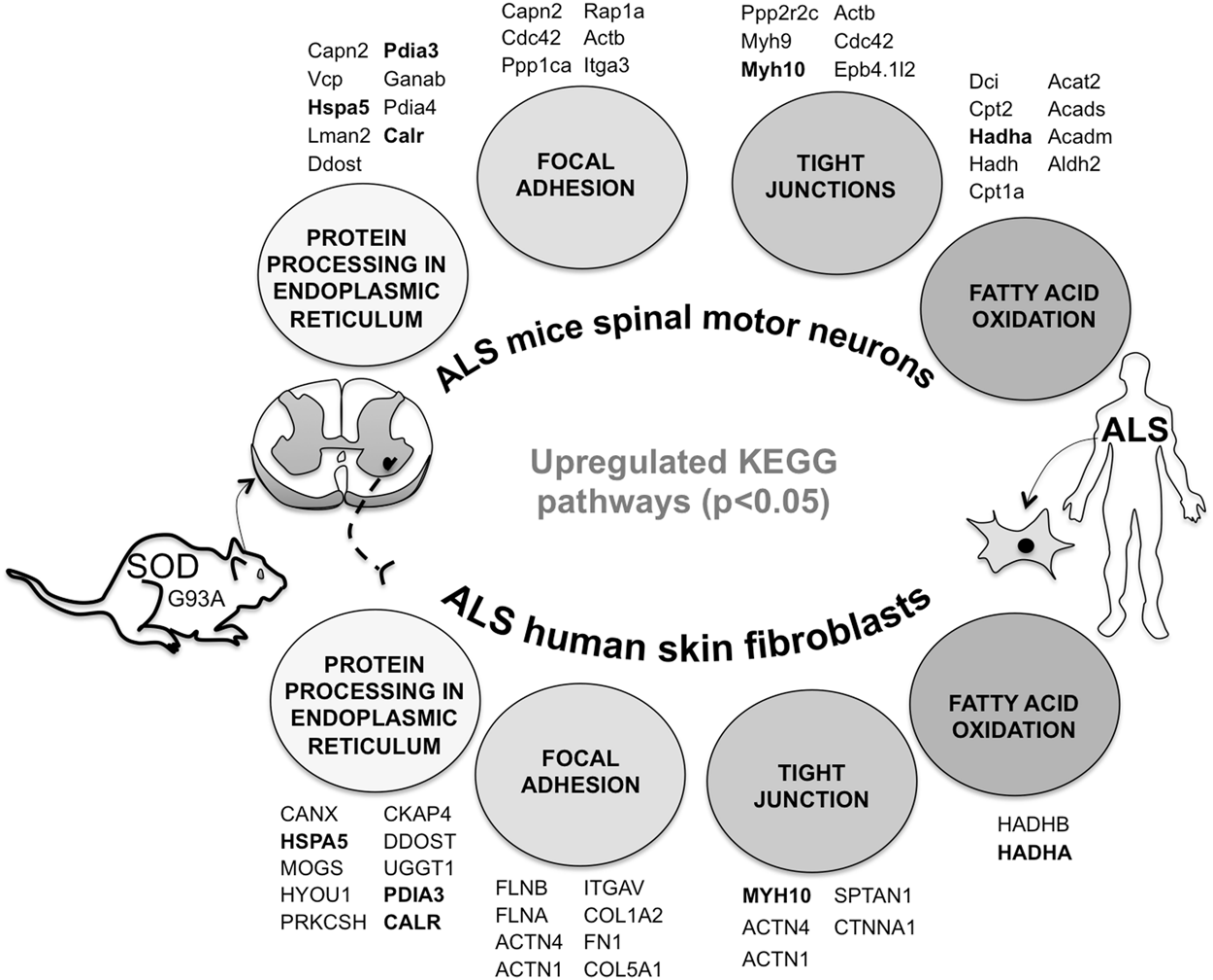
Common pathogenic proteomic features of Amyotrohpic Lateral Sclerosis between SOD1G93A mice motor neurons and skin fibroblasts. The KEGG pathway significantly enriched (FDR < 0.05) in the dataset of proteins over-expressed in SODG93A mice motor neurons (versus WTs) are illustrated on top of the figure and the corresponding proteins are also shown. Similar analysis performed in ALS patients skin fibroblasts is shown on the bottom of the figure. The proteins over-expressed in both models are shown in bold.

Interestingly, our work enabled to highlight that skin fibroblasts from ALS sporadic patients and motor neurons from SODG93A mice share common molecular and functional features (Fig. 6): *i)* OXPHOS impairment, *ii)* increased HADHA expression and *iii)* reduced expression of specific ribosomal proteins. However, one main difference resides in that motor neurons stimulate numerous energy-producing pathways including FAO but also glycolysis, while in skin fibroblasts glucose metabolism is reduced as well as specific steps of FAO. The predicted main regulators of proteome remodeling also differ between the two models, supporting the view of a tissue-specific alteration in ALS pathophysiology. Lastly, our findings of a proteomic signature with increased expression of oxidative phosphorylation systems in ALS mouse motor neurons suggests that preclinical pharmacological approaches aiming to stimulate mitochondrial respiration could be tested. The oral administration of coenzyme Q_10_, a respiratory chain intermediate but also an antioxidant, significantly increased the life span in a transgenic mouse model of familial amyotrophic lateral sclerosis^55^. However, a clinical trial (identifier NCT00243932) performed on 75 ALS patients receiving 2700 mg of CoQ_10_ daily *versus* placebo showed no significant difference (p = 0.14) in the ALS Functional Rating Scale-revised (ALSFRSr) Score after 9 months. Other trials using creatine also failed to show any benefit of creatine on any outcome measure^56^. Still, no specific therapeutic approach aiming at the restoration of mitochondrial function or the reduction of neuronal ketoacidosis has been tested in humans, and additional preclinical studies are needed in that field. In particular, our observations suggest that the pharmacological stimulation of fatty-acid oxidation is risky, unless ketoacidosis can be avoided. Our work provides a unique set of data that will inform future studies that aim to further investigate and define the impact of neurometabolic and proteostatic defects on ALS pathology as well as to propose and evaluate innovative preclinical metabolic therapeutic strategies.

Certainly, our work has limitations in the study design, the impact and the statistics. First, we focused our investigations on motor neuron bioenergetics and proteomics, but several analyses showed that ALS pathophysiology also depends on glial cell molecular and functional alterations. In particular, neuronal bioenergetics is strongly connected to astrocytes^57^, and our cell culture procedures remove the metabolic exchanges between neurons and glial cells evidenced *in vivo*. Therefore, co-culture systems would be required to better investigate the alterations in metabolic symbiosis in ALS. We also performed our analyses on mice motor neurons adapted to artificial cell culture conditions, and the composition of the media as well as the exposure to atmospheric partial pressure of oxygen might have modified both the bioenergetics and the proteome^58^.

To confirm our hypothetical model of ‘adaptive FAO’, additional metabolomic investigations using [U^13^C]-palmitate might be necessary to validate the metabolic role of UCP2 as a gatekeeper between the entry of oxaloacetate in the TCA on one hand and ketogenesis and cholesterol synthesis on the other. The metabolic alterations observed in ALS patients’ skin fibroblasts may not recapitulate what occurs in the spinal cord due to the tissue-specific regulation of energy metabolism^59^. Moreover, All patients cells used in our study were tested for C9ORF72, SOD1, FUS, TDP43 mutations but none of them carried any mutation in these genes. In addition, disease duration could be considered as a potential confounder in the analysis on human skin fibroblasts since most patients were quite long-lived. Most notably, the ALS mouse model used in our study, the SODG93A mutant mice, as well as the patients’ skin fibroblasts do not reflect the variety of ALS genetics and pathophysiology. The SOD1G93A mice have additional limitations, as extensively discussed by Philipps and Rothstein^60^.

Finally, our work provides a unique set of data to settle and guide further investigations to define the impact of neurometabolic defects on ALS pathology as well as to propose and evaluate innovative preclinical therapeutic strategies. However, the possibility of exploring whether HADHA, ACAT2 or UCP2 are reliable biomarkers of ALS will require validation studies on a larger number of samples, ideally freshly excised human tissues.

## Methods

### Chemicals

Reagents were purchased from Sigma, at the exception of the ATP/ADP monitoring kit (Abcam) and the primary antibodies (see the westernblot section below).

### Culture of mice motor neurons and patient skin fibroblasts

Motor neurons were obtained from the dissociation of embryonic ventral spinal cords from 12.5 days SOD1-G93A (ALS) and “wild type” control littermate (heterozygous) mice embryos (litters from heterozygous females coupled to homozygous SOD1-G93A males), as described in^61^. Briefly, the entire spinal cords were dissected and regrouped by genotype. The dorsal root ganglia and meninges were withdrawn as well as the dorsal part of the spinal cord. The remaining ventral spinal cords were chopped and dissociated with trypsin, and cells were separated by density gradient centrifugation through OptiPrep Density gradient medium. The fractions containing motor neurons were carefully collected and filtered through a BSA cushion, and the purified motor neurons seeded on poly-D-lysine- and laminin-coated culture dishes. Cultures were maintained in Neurobasal medium supplemented with 2% B27, 2% inactivated horse serum, 100 U/ml penicillin and 100 U/ml of streptomycin, and with glutamate, glutamine, and neurotrophic factors (GDNF 10 μg/ml, BDNF 1 μg/ml, CTNF 1 μg/ml), in 5% CO_2_ at 37 °C. For all the experiments, cells were analyzed between DIV 3 and DIV 5 to ensure minimal glial invasion. The term ‘DIV’ is widely used in the field of neurosciences when referring to primary neurons in culture and means ‘Days *in vitro*’. The method to analyze neuronal cell death after different metabolic treatments was adapted from Szelechowski *et al*.^62^. Briefly, cultures were stained with SMI32 and DAPI and the number of pyknotic nuclei within the SMI32 positive cells were manually assessed on 3 pictures randomly taken for each culture case (20x, open field fluorescence microscope, Leica). At least 100 neurons were analyzed per case and per experiment, on 3 independent experiments.

Primary skin fibroblasts were collected from the arm of ALS patients and control age and gender-matched subjects. All patients were recruited at the reference center for ALS at the hospital of Bordeaux and gave written informed consent to participate in this study, according to the Declaration of Helsinki. The cells were grown in Dulbecco’s Modified Eagle Media (DMEM) containing 5 mM glucose and supplemented with 15% fetal bovine serum, 100 U/ml penicillin and 100 U/ml streptomycin, in 5% CO2 at 37 °C. For all experiments, the cells were harvested during the exponential phase of growth at 70% confluency. The primary fibroblasts cell lines were generated on the same day and the cells underwent the same number of passages (P) at the moment of the various experiments (P4 to P6).

### Bioenergetic measurements

Respiration of motor neurons was analyzed on the XF96 analyzer (SeaHorse Biosciences) in XF assay medium while respiration of the skin fibroblasts was evaluated using high-resolution respirometry (Oroboros). The term ‘routine’ respiration is defined as the respiratory rate of intact cells measured in 5 mM glucose DMEM under atmospheric conditions at 37 °C and sensitive to 2.5 µM antimycin A inhibition. The term ‘oligo’ respiration is the respiratory rate measured in the routine conditions after addition of the F_1_F_0_ATPsynthase inhibitor oligomycin at 20 µg/ml. This ‘oligo’ state of respiration does not depend on ADP phosphorylation. The term ‘FCCP’ respiration defines the rate of respiration measured in the ‘oligo’ conditions after addition of the uncoupler FCCP used at 2 µM. The ‘FCCP’ state allows to evaluate the maximal capacity of the respiratory chain in presence of energy substrates and oxygen concentration as defined in the ‘routine’ conditions. The ‘routine/oligo’ ratio allows to quantify the coupling degree of the respiratory chain with ADP phosphorylation in the routine conditions. The FCCP/Routine ratio gives a measure of the capacity of the respiratory chain to be chemically uncoupled. It indicates how far from the maximal capacity the routine respiration operates. To assay motor neurons bioenergetics we used the SeaHorse extracellular flux analyzer (XF96). For each run we plated 10.000 cells per well in a 96 well-plate. Each condition was replicated in 3–5 wells (technical replicates) and this experiment was repeated 3 times (biological replicates). The rate of respiration was expressed per 10.000 cells. The primary culture of mouse motor neurons was obtained as follow: typically, one culture dish was obtained from 5 different littermate embryos (pooled). So, the biological replicates correspond to different cultures, each of those obtained from 5 animals.

To assay the ALS patient’s derived primary skin fibroblasts we used the Oroboros system. For each run 1 million cells per milliliter of cell culture medium was used. Three technical replicates were performed for three different cultures (biological replicates; N = 3). The rates of respiration were normalized per million of cells. The intracellular ATP content of motor neurons was measured using the bioluminescent ATP determination kit (Molecular Probes, Life Technologies). 20 uL of a cell suspension of 5 × 10^5^ SOD1G93A or wild type motor neurons/mL were plated in a 96-well plate. On DIV 5, cells were treated or not with genipin (20 μM, 45 min), lysed using the lysis buffer provided with the kit (30 μl/well) and the lysates were immediately transferred into annotated tubes kept in ice and protected from light. ATP concentration was determined by the light-emitting luciferase-catalyzed oxidation of luciferin with ATP and bioluminescence measurement on a luminometer (Luminoskan, Labsystems, Finland). Standardization was performed using known quantities of standard ATP provided with the kit. Determination of the mitochondrial transmembrane electric potential in cultured mice motor neurons was performed by fluorescence imaging using tetramethylrhodamine methyl ester (TMRM). Briefly, cells were incubated with 20 nM TMRM during 20 min. at 37 °C and confocal fluorescence microscopy was performed on a Zeiss Axio Observer microscope equipped with the Vivatome system with a 63x oil spring loaded objective. The images were acquired using AxioVision (6D acquisition and vivatome modules). A minimum of 30 different cells from three different experiments were selected randomly per experimental condition, and the analysis of the fluorescence signal intensity was performed using Morphostryder (Explora Nova).

### Mitochondrial morphology analysis

Mitochondria length was measured in all neurites of individualized SOD and WT motor neurons. Mitochondrial network was revealed by Tom20 (mitochondrial outer membrane protein) staining. Mitochondria length was measured and recorded using ImageJ software as described in^62^. For each neuron, mitochondrial tubules were ranked by size categories (short: <2uM; intermediate: 2–6uM; long: >6 uM). We analyzed 20 motor neurons per culture, each obtained from 3 to 5 different littermate embyos of each genotype, 3 independant cultures were performed for this analysis.

### Proteomics and westernblot

The steps of sample preparation and protein digestion, nLC-MS/MS, database search and results processing, and label-free quantitative data analysis were described in^63^. Briefly, peptide mixture was analyzed on a Ultimate 3000 nanoLC system (Dionex) coupled to a nanospray LTQ-Orbitrap XL mass spectrometer (ThermoFinnigan, San Jose, CA). Data were searched by SEQUEST through Proteome Discoverer 1.4 (Thermo Fisher Scientific Inc.). Raw LC-MS/MS data were imported in Progenesis LC-MS 4.0 (Nonlinear Dynamics Ltd, Newcastle, U.K) and data processing included the following steps: (i) Features detection, (ii) Features alignment across the 12 sample, (iii) Volume integration for 2–6 charge-state ions, (iv) Normalization on total protein abundance, (v) Import of sequence information, (vi) ANOVA test at peptide level and filtering for features p < 0.05, (vii) Calculation of protein abundance (sum of the volume of corresponding peptides), (viii) ANOVA test at protein level and filtering for features p < 0.05. Noticeably, only non-conflicting features and unique peptides were considered for calculation at protein level. Pathway analysis was performed using IPA (Qiagen), KEGG (String) and Panther. Western blotting was performed as described in^63^. In this study we used the following Abcam antibodies: SOD1 (ab13498), SOD2 (ab13533) and catalase (ab16731).

### ROS production and glutathione REDOX state determination

The cells were trypsinized and counted to obtain a cell suspension of 1 × 10^6^ cells/mL. Changes in cytosolic H_2_O_2_ levels were monitored using the CM-H_2_DCFDA probe. The probe was added to the cell suspension and incubated for 30 min at 37 °C, according to the manufacturer’s protocol. The cells were then washed twice in PBS, and fluorescence was measured in a quartz cuvette on a Xenius spectrofluorometer (SAFAS, Monaco, France). A second reading was performed with the addition of 100 µM H_2_O_2_ in the cuvette to verify the response and absence of saturation of the CM-H_2_DCFDA probe. The signal increased immediately after the addition of H_2_O_2_ in a dose-dependent manner. To evaluate the ratio of GSH/GSSG we used the detection assay kit from Abcam (ab138881) following manufacturer’s protocol.

### Statistical analysis

All of the data presented in this study correspond to the mean value of n experiments ± SD, with a minimum of n ≥ 3. Comparison of the data sets was performed with either the Student’s t-test, or the two-way ANOVA test including Sidak’s multiple comparison using Prism 7 (Graphpad Software). Two sets of data were considered statistically different when P < 0.05. Prior to perform unpaired t test we verified the Gaussian distribution of the data by performing a normality test (Prism 7, GraphPad Software).

### Statement

The use of human tissue samples was approved by the University Hospital Center (CHU) of Bordeaux and skin biopsies were obtained at the reference center for ALS at the CHU. Each patient gave written informed consent to participate in this study, according to the Declaration of Helsinki. Moreover, all experiments and methods were carried out in accordance with relevant guidelines and regulations and all experimental protocols were approved by the CHU.

## Electronic supplementary material

Supplementary information

Supplementary Dataset 1

Supplementary Dataset 3

Supplementary Dataset 4

Supplementary Dataset 5

Supplementary Dataset 6

Supplementary Dataset 7

Supplementary Dataset 2
