# Supplementary material for: Metabolic Reprogramming in Amyotrophic Lateral Sclerosis
Source: Sci Rep. 2018 Mar 2;8:3953. doi: 10.1038/s41598-018-22318-5 (PMC5834494; doi:10.1038/s41598-018-22318-5)
Supplement: Supplementary file 1 — Supplementary information [file 41598_2018_22318_MOESM1_ESM.docx]

**METABOLIC REPROGRAMMING IN**

**AMYOTROPHIC LATERAL SCLEROSIS**

Szelechowski M.^1,2§^, Amoedo N.^2,3§^, Obre E.^4§^, Léger C.^1,2^, Allard L.^1,2^, Bonneu M.^5,2^, Claverol S.^5,2^, Lacombe D.^2,3^, Oliet S.^1,2^, Chevallier S.^1,2^, Le Masson G.^1,2#*^, Rossignol R.^2,3#*^.

**Supplementary information :** legends of the Supplementary Dataset files (supplementary datasets include two Figures as .TIFF files and 7 Tables as separate Excel documents).

**Figure S1:** Top two protein networks significantly different in ALS mouse motor neurons. (**A**) ‘protein synthesis, protein degradation cellular assembly and organization’ (score 38)’ and (**B**) ‘lipid metabolism, small molecule biochemistry, energy production’ (score 28).

**Figure S2**: Downstream targets network of MYC and RICTOR identified in the proteome differences between skin fibroblasts from ALS patients or healthy individuals.

**Table S1:** List of the cellular functions and related diseases identified by the IPA in ALS mouse motor neurons.

**Table S2:** KEGG pathway analysis of the proteins over-represented in ALS motor neurons proteome.

**Table S3:** KEGG pathway analysis of the proteins under-represented in ALS motor neurons proteome.

**Table S4 :** Clinical details of the ALS patients used to obtain skin fibroblasts. Ketonuria was measured semi-quantitatively using a urine dipstick test. Patient 3 is listed in the Table as it was considered for the ketonuria analysis along with P1, P2, P4 and P5. However, for all the other measurements we used P1, P2, P4 and P5 cells.

**Table S5 :** List of the Ingenuity Pathways significantly altered in the proteome of ALS patients skin fibroblasts.

**Table S6 :** List of the predicted regulators of the protemic changes observed in ALS mice motor neurons (IPA).

**Table S7 :** Detail of the metabolic proteins overexpressed or underexpressed in ALS patients skin fibroblasts (P<0.05 ; at least 2 proteins per pathway).


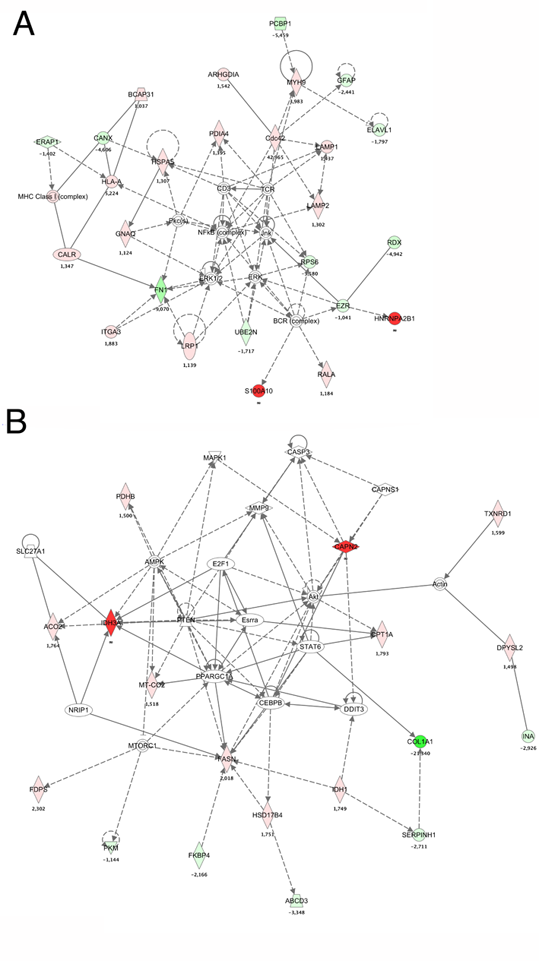


**Figure S1:** Top two protein networks significantly different in ALS mouse motor neurons. (**A**) ‘protein synthesis, protein degradation cellular assembly and organization’ (score 38)’ and (**B**) ‘lipid metabolism, small molecule biochemistry, energy production’ (score 28).


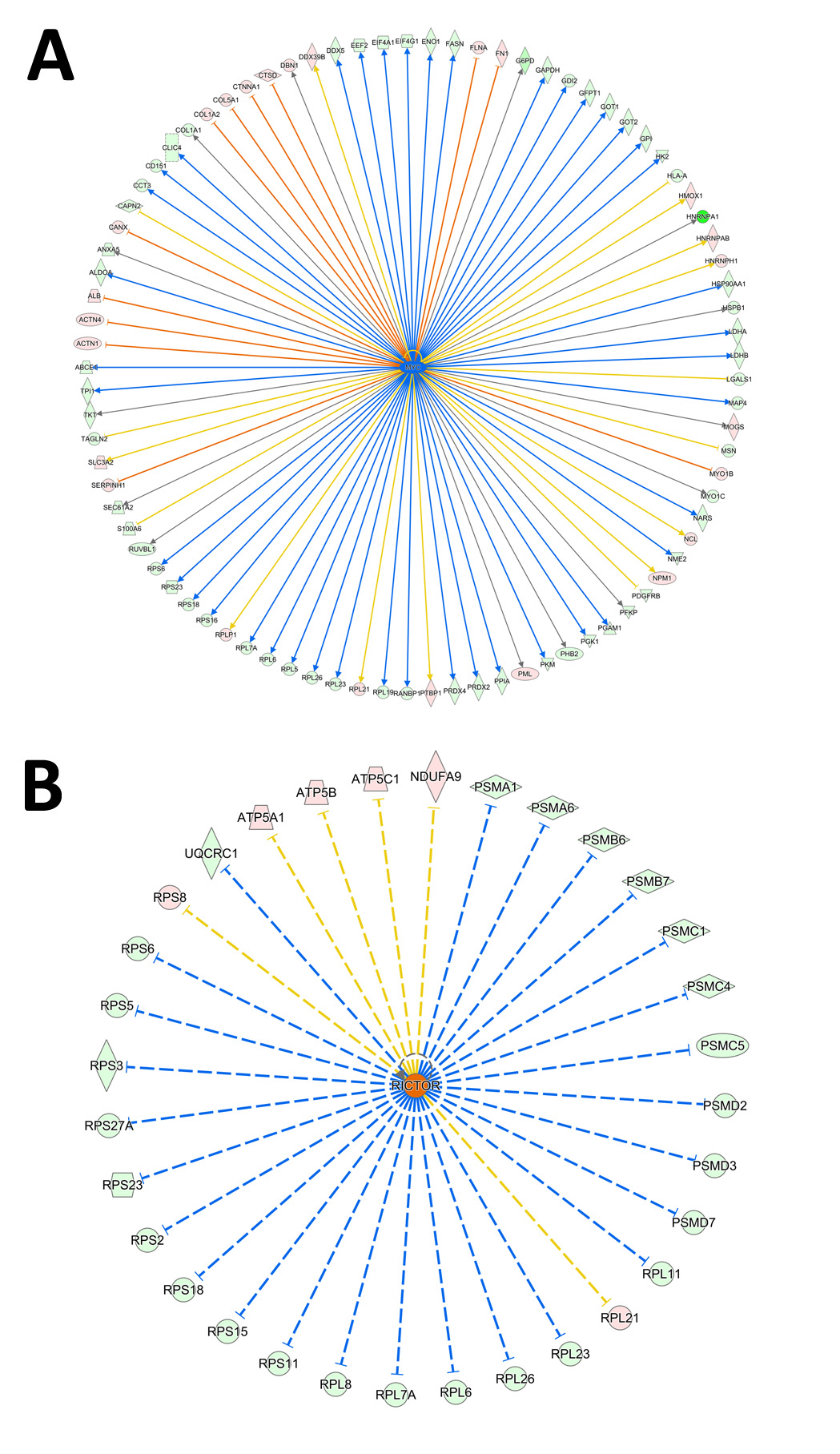


**Figure S2**: Downstream targets network of MYC and RICTOR identified in the proteome differences between skin fibroblasts from ALS patients or healthy individuals.
